# A Meta-Analysis of eHealth Interventions on Ischaemic Heart Disease Health Outcomes

**DOI:** 10.5334/gh.1173

**Published:** 2023-03-16

**Authors:** Puteri Sofia Nadira Megat Kamaruddin, Azmawati Mohammed Nawi, Mohd Rizal Abdul Manaf, Mohamad Nurman Yaman, Abdul Muizz Abd Malek

**Affiliations:** 1 Department of Community Health, Universiti Kebangsaan Malaysia, Kuala Lumpur, MY; 2 Department of Medical Education, Universiti Kebangsaan Malaysia, Kuala Lumpur, MY; 3 Cardiology Department, Hospital Serdang, Kajang, MY

**Keywords:** eHealth intervention, ischaemic heart disease, meta-analysis, LDL

## Abstract

**Background:**

Electronic Health (eHealth) interventions as a secondary prevention tool to empower patients’ health in decision-making and behaviour.

**Objective:**

With the growing body of evidence supporting the use of eHealth interventions, the intention is to conduct a meta-analysis on various health outcomes of eHealth interventions among ischaemic heart disease (IHD) patients.

**Methods:**

Based on PRISMA guidelines, eligible studies were searched through databases of Web of Science, Scopus, PubMed, EBSCOHost, and SAGE (PROSPERO registration CRD42021290091). Inclusion criteria were English language and randomised controlled trials published between 2011 to 2021 exploring health outcomes that empower IHD patients with eHealth interventions. RevMan 5.4 was utilised for meta-analysis, sensitivity analysis, and risk of bias (RoB) assessment while GRADE software for generating findings of physical health outcomes. Non-physical health outcomes were analysed using SWiM (synthesis without meta-analysis) method.

**Results:**

This review included 10 studies, whereby, six studies with 895 participants’ data were pooled for physical health outcomes. Overall, the RoB varied significantly across domains, with the majority was low risks, a substantial proportion of high risks and a sizeable proportion of unclear. With GRADE evidence of moderate to high quality, eHealth interventions improved low density lipoprotien (LDL) levels in IHD patients when compared to usual care after 12 months of interventions (SMD –0.26, 95% CI [–0.45, –0.06], I^2^ = 0%, p = 0.01). Significance appraisal in each domain of the non-physical health outcomes found significant findings for medication adherence, physical activity and dietary behaviour, while half of the non-significant findings were found for other behavioural outcomes, psychological and quality of life.

**Conclusions:**

Electronic Health interventions are found effective at lowering LDL cholesterol in long-term but benefits remain inconclusive for other physical and non-physical health outcomes for IHD patients. Integrating sustainable patient empowerment strategies with the advancement of eHealth interventions by utilising appropriate frameworks is recommended for future research.

## 1.0 Introduction

Ischaemic heart disease (IHD) persists as a major contributor to premature mortality and death rates worldwide, with economic growth and urbanisation exerting the greatest influence on its onset [1]. Socioeconomic changes, increased life expectancy, and lifestyle-related risk factors have all contributed to the increased IHD mortality in recent decades [2]. Patients with IHD have a greater risk of premature death, myocardial infarction, and readmission. Following diagnosis, international guidelines advocate the implementation of secondary prevention strategies [3]. These strategies include physical activity, lifestyle modification guidance, symptom and medication management, and psychosocial support to improve IHD outcomes [4]. The World Health Organization stated that risk factor modification and self-care can prevent approximately 80% of cardiac events [5].

Secondary prevention through electronic health (eHealth) interventions is a viable substitute for conventional cardiac rehabilitation as they can be implemented immediately. Despite being established in the 1990s, the term ‘eHealth’ did not become widely used until 1999 [6]. Electronic Health, telemedicine, and mobile Health (mHealth) are frequently used interchangeably. Although there are differences between the concepts, it is now increasingly common to use eHealth as a blanket term that includes telemedicine and mHealth [7]. A new discipline at the nexus of medicine, public health, and business, eHealth is defined as the improvement of health-related information and services via electronic means [6]. For the purpose of this research, the umbrella term ‘eHealth’ refers to health-related information and communication technologies, such as smartphone mobile applications (apps), short message service (SMS), websites, emails, telemonitoring, phone calls, and wearables/monitoring devices (pedometer, accelerometer, smartwatch, sleep tracker, heart rate monitors) [8]. Nevertheless, there is limited evidence evaluating the health outcomes of eHealth interventions among patients with IHD.

In particular, eHealth interventions benefit from the vast functionalities of new technologies that enable people to access health information and educational content quickly and easily to continuously monitor their health status and behaviour and to receive individualised feedback about the suitability of their actions and physiological parameters in real-time. Additionally, eHealth interventions enable patients to easily communicate online with their caregivers and other patients. All of these characteristics may be very important for encouraging and assisting people in choosing and maintaining healthy lifestyles, which will subsequently inhibit disease onset or progression. Finally, gamification helped eHealth treatments better encourage people to adhere to long-term preventative and lifestyle change interventions. For all of these reasons, using eHealth interventions is a promising strategy to improve the health of IHD patients.

Previous research in this field focused primarily on telehealth interventions, which are defined as healthcare delivery over the phone, the Internet, or videoconferencing [9,10]. Telehealth is more hospital-based or clinician-dependent, where the hurdle of resource utilisation in terms of requiring skilled experts for treatment delivery and health budget may also occur with current individualised and patient-centred eHealth interventions. Despite the aforementioned issue, eHealth intervention accessibility has evolved and is available according to individual affordability. Websites, mobile apps, email, mobile phones, SMS, and monitoring sensors are all current common communication modes. These modalities enable eHealth interventions to encourage healthy behaviours in real-time, enabling users to access and interact with data, upload and review records, receive automated feedback, and communicate with peers or healthcare professionals [11]. Individualised eHealth interventions can accommodate individual risk factors, care needs, objectives, and resources to change health behaviour [12]. Approximately 65.6% of the world population uses the Internet [13], and older adults are increasingly using it as an important health information source and a patient empowerment tool in health decision-making and behaviour [14].

Thus, there is growing support for delivering secondary prevention care components via the eHealth platform that best address cardiac patients’ individualised healthcare needs, resulting in additional health benefits and identifying impediments to service access and use [15]. Based on the growing body of evidence supporting the use of eHealth interventions, a meta-analysis was conducted on the health outcomes of eHealth interventions among patients with IHD.

## 2.0 Methods

### 2.1 Design

This review followed the procedures outlined in the Cochrane Handbook for Systematic Reviews [16] (The Cochrane Collaboration, Oxford, UK) of Interventions and reported using the Preferred Reporting Items for Systematic Reviews and Meta-Analyses (PRISMA) statement [17]. The PRISMA protocol encourages researchers to obtain accurate information from reliable sources. The systematic literature review was planned using this protocol by developing an appropriate research question. The PRISMA Checklist can be found in Supplementary Document 1 and 2. The systematic search was classified into three stages: identification, screening, and inclusion. PROSPERO had registered the systematic review (CRD42021290091), and the study protocol can be accessed at https://www.crd.york.ac.uk/prospero/displayrecord.php?RecordID=290091.

### 2.2 Search methods

A comprehensive search was conducted across five databases, including Web of Science, Scopus, PubMed, EBSCOHost, and SAGE, covering the 10-year period from 2011 to 2021 in light of recent innovations in the advancement of eHealth interventions. The PICO framework was utilised to identify keywords that aided authors in formulating a fundamental research question. It was characterised by three concepts: Population or Problem, Interest or Intervention, and Outcome. The formulated keywords based on these concepts are ‘Adult’ and ‘Ischaemic Heart Disease’ (Population), ‘eHealth intervention’ (Intervention) and ‘outcome’ (Outcome), which served as the basis for the formulation of the research objective. Comparator element was not defined in this study since there was no comparator to be examined and it was not part of the study’s design to compare the health outcomes against those of eHealth interventions.

All searches were conducted within a week of the 1^st^–7^th^ December 2021. The identification stage included a search for all possible synonyms, medical subject heading (MeSH) terms, similar terms, and variants of the keywords: ‘Adult’, ‘Ischaemic Heart Disease’, ‘eHealth intervention’ and ‘outcome’ together with the Boolean operators (Supplementary Document 3). This method provided better coverage for locating relevant articles in the selected databases. These databases were distinguished by their extensive literature collections and advanced search features.

### 2.3 Search outcome

The database search revealed 1231 English language articles published between 2011 and 2021. After 87 duplicates were removed, 1144 articles were further screened with the following criteria to determine inclusion: (1) randomised controlled trials (RCTs); (2) IHD patients aged 18 and above were recruited; (3) made use of a website or a mobile apps in addition to other ways of communicating (email, SMS, phone call) and (4) provided information about physical and non-physical health outcomes (behavioural, psychological, quality of life (QoL) and others). This meta-analysis included only RCTs to generate the most robust evidence for eHealth interventions [18]. The type of study that explicitly stated not randomised, quasi-experimental, pre-post, review articles, editorials, proceedings and commentary articles were excluded.

**Figure 1:**
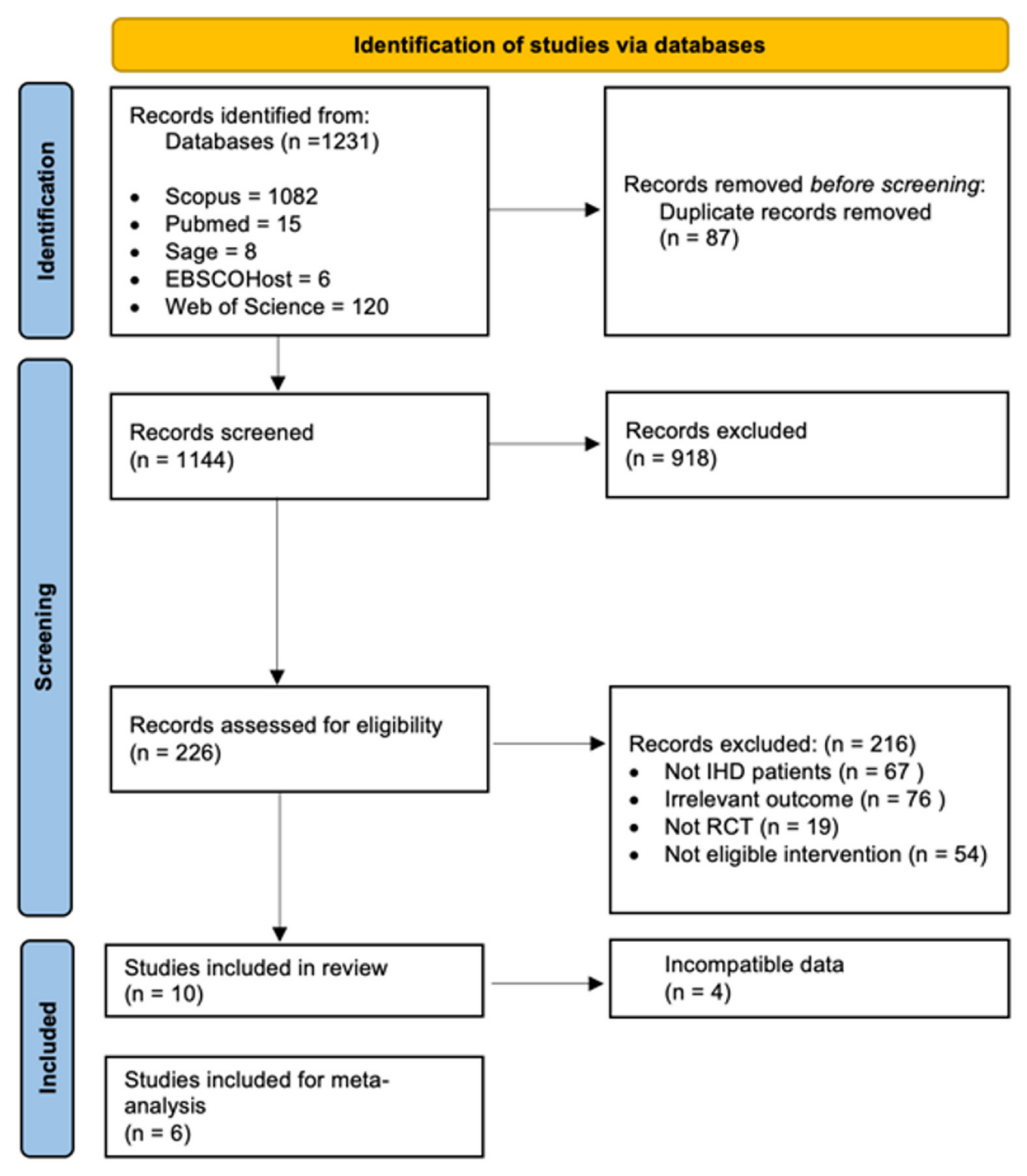
PRISMA diagram of identifying studies of eHealth interventions [17].

This process resulted in manually sorting 226 articles that concentrated on participants who were directly connected to the Internet and actively used it, with or without the assistance of other mechanisms, rather than relying solely on data transfer via wearable monitors between patients and professionals. Comparative studies included those in which participants received no intervention, standard or usual care from their health care systems. This review omitted articles in which participants were monitored exclusively via devices or received SMS or phone call reminders without the use of Internet. This method resulted in the exclusion of 216 articles based on unsuitable target population, not RCT and irrelevant health outcomes, e.g., medication trial and merely one-way monitoring patients with eHealth interventions. Final eligibility process included 10 articles [19,20,21,22,23,24,25,26,27,28]. However, only six [19,20,22,24,25,27] were fit for meta-analysis due to data compatibility. Figure 1 depicts the PRISMA flow for study identification.

Overall, two independent reviewers (PSNMK and AMN) conducted a sequential review and selection of studies, removed duplicates and evaluated the eligibility of the abstract to the full text. Any disagreements about the study’s inclusion were resolved through consultation with a third investigator (MRAM). The data was transferred to Review Manager 5.4 software [29] by one review author (MNY) and a second reviewer (AMAM) verified the accuracy of the data entry.

### 2.4 Assessment of risk-of-bias and certainty of evidence rating

The Cochrane risk of bias (RoB) tool for randomised trials (RoB 2) was used to assess the study quality as it is the most frequently used tool for randomised trials [30]. This tool is an outline for determining the RoB in a single outcome (an estimate of the effect of an experimental intervention compared to a comparator on a specific outcome) from any type of randomised trial. Within each domain, a series of key questions were used to elicit information about trial characteristics that are associated with bias risk. The following seven major criteria were used by two review authors (PSNMK and MNY) to independently assess each included trial for RoB: random sequence generation, allocation concealment, blinding of participants and personnel, blinding of outcome assessment, incomplete outcome data, and selective reporting and other bias. Each domain was classified as having a low, moderate, or high RoB. If there was disagreement, a third author (MRAM) led a discussion leading to a consensus. Meanwhile, the ‘Summary of findings’ table contains the certainty of evidence determination from meta-analysis outcome using the well-established and widely used Grading of Recommendations Assessment, Development and Evaluation (GRADE) method (Supplementary Document 4) [31].

### 2.5 Assessment of heterogeneity, meta-analysis

When three or more studies reported the same outcome, the Review Manager 5.4 was used to pool the data; otherwise, synthesis without meta-analysis (SWiM) was performed [32]. SWiM checklist can be found in Supplementary Document 5. The I^2^ statistic was used to measure the degree of heterogeneity in the results with a 50% threshold deemed significant [33]. Data were pooled and meta-analysis performed where necessary, using the RevMan 5.4 software’s random-effect model [29], as the assumption was there might be a significant effect between the studies in that they differ in terms of population and sample sizes. The findings were presented using the standard mean difference (SMD) for continuous outcomes and their respective 95% confidence intervals (CI) because different methods were utilised to measure the same outcome and a study [24] result needs to be converted to produce the same measures. To explore the differences, subgroup analysis was employed. When the pooled effect displayed a high level of heterogeneity, a sensitivity analysis was conducted [16]. Publication bias was assessed by using funnel plots.

## 3.0 Results

There were 10 RCTs included in this review published between 2015 and 2020. The characteristics of the included studies were summarised in Table 1, and the overall modality of the various eHealth interventions utilised to measure different health outcomes was summarised in Table 2. The number of participants ranged from 48 [21] to 312 [25].

**Table 1:** Characteristics of reviewed studies (n = 10).


AUTHOR, YEAR	COUNTRY	PARTICIPANTS MEAN AGE (SD)	SAMPLE SIZE	STUDY DURATION (FOLLOW UP)	INTERVENTION GROUP	CONTROL GROUP	HEALTH OUTCOMES			

							PHYSICAL	NON-PHYSICAL		

								BEHAVIOURAL	PSYCHOLOGICAL	OTHERS

Dale 2015 [19]	New Zealand	59.5 (11.1)	123	10 months	Guidelines delivered by SMS and a supporting website over 24 weeks to educate patients about cardiovascular risk factors and support to make relevant lifestyle changes. Recommended lifestyle changes included stopping smoking, limiting alcohol consumption, eating right and maintaining regular physical activity	Usual care	BMI, BP, lipid profile	Medication adherence, physical activity, health behaviour	Anxiety, Depression	–

Frederix 2015 [20]	Belgium	61 (9)	140	1 year 7 months	24-week telerehabilitation programme in combination with conventional cardiac rehabilitation	Conventional cardiac rehabilitation	BMI, BP	Physical activity	–	QoL

Martin 2015 [21]	United States of America	58 (8)	48	4 months	Receiving SMS	Usual care	–	–	–	–

Skobel 2017 [22]	Spain, United Kingdom and Germany	*IG: 60 (50/65)*CG: 58 (52/67)	118	1 year 10 months	GEX, sensor monitor breathing rate, ECG	Conventional care	BMI, BP, lipid profile	-	Anxiety, depression	QoL

Kamal 2018 [23]	Pakistan	IG: 59.1 (11.6)CG: 57.7 (11.1)	197	9 months	Access to helpline number to address queries in addition to standard of care as per institutional guidelines, IVR technology tailored to their respective prescriptions and have the ability to hear information about medication dosage, correct use, side effects, mechanism of action and how and why use the medication. Also receive scheduled SMS message reminders to take their medications.	Regular follow-up visits with stroke neurologist or cardiologist	–	Medication adherence	–	–

Choi 2019 [24]	American	IG: 57.2 (1.8)CG: 56.6 (1.7)	100	6 months	Custom smartphone application that reinforced the Mediterranean diet	In-person dietary counselling sessions with registered dietitian at 1 month and 3 months	BMI, BP, lipid profile	Diet	–	–

Dorje 2019 [25]	China	IG: 59.1 (9.4)CG: 61.9 (8.7)	312	4 months (FU-1 year)	Standard care and 2-month intensive SMART-CR/SP programme, followed by a 4-month step-down stage. During the intensive phase, participants received four educational modules per week via WeChat. In the step-down phase, participants received only 2 cartoon pictures with key motivational message per week.	Standard care provided by community doctors and cardiologists (brief inpatient health education by ward nurse, medication management, and ad-hoc follow-up visits to a cardiologist or other health-care providers according to the patient’s self-assessment of their cardiovascular health). WeChat for sending follow-up visit reminders	BMI, BP, lipid profile	Medication adherence, physical activity, dietary behaviour, smoking cessation	–	QoL

Broers 2020 [26]	Netherlands and Spain	61.97 (11.61)	150	5 months (FU-6 months)	Ambulatory health-behaviour assessment technologies for 6 months combined with a 3-month behavioural intervention programme	Usual care	–	Health Promotion Lifestyle Profile	–	QoL

Lunde 2020 [27]	Norway	59 (8.7)	113	9 months (FU-1 year)	Individualised follow-up enabled with an application for one year	Usual care	BP, lipid profile	–	–	QoL

Song 2020 [28]	China	IG: 54.17 (8.76)CG: 54.83 (9.13)	106	6 months	Using telemonitoring software (MEMRS-CRS, developed by Medicus) on smartphones, heart rate monitors (Suunto, provided by Medicus) for monitoring patients’ HR	Usual care	–	Physical activity	–	–


SD: standard deviation, IG: intervention group, CG: control group, FU: follow up post-intervention, HR: heart rate, BP: blood pressure, BMI: Body Mass Index, SMS: Short Message Service, QoL: Quality of Life, IVR: Interactive Voice Response, GEX: Guided exercise system, ECG: Electrocardiogram, SMART-CR/SP: smartphone-based and WeChat-based cardiac rehabilitation and secondary prevention. * Median (25th/75th centile).

**Table 2:** Overview of the overall modality of the eHealth interventions (n = 10).


AUTHOR, YEAR	EHEALTH INTERVENTIONS MODALITY						

	SMARTPHONE MOBILE APPLICATION	SMS	WEBSITE	EMAIL	TELEMONITORING	PHONE CALL	OTHER DEVICES

Dale 2015 [19]		/	/				Pedometer

Frederix 2015 [20]		/		/	/		Accelerometer

Martin 2015 [21]	/	/	/				Accelerometer

Skobel 2017 [22]	/				/		

Kamal 2018 [23]		/			/	/	

Choi 2019 [24]	/						

Dorje 2019 [25]	/	/					

Broers 2020 [26]	/	/				/	Smartwatch, sleep tracker

Lunde 2020 [27]	/						

Song 2020 [28]	/	/			/	/	Heart rate monitors


SMS: Short Message Service.

### 3.1 Risk-of-bias in included studies

**Figure 2:**
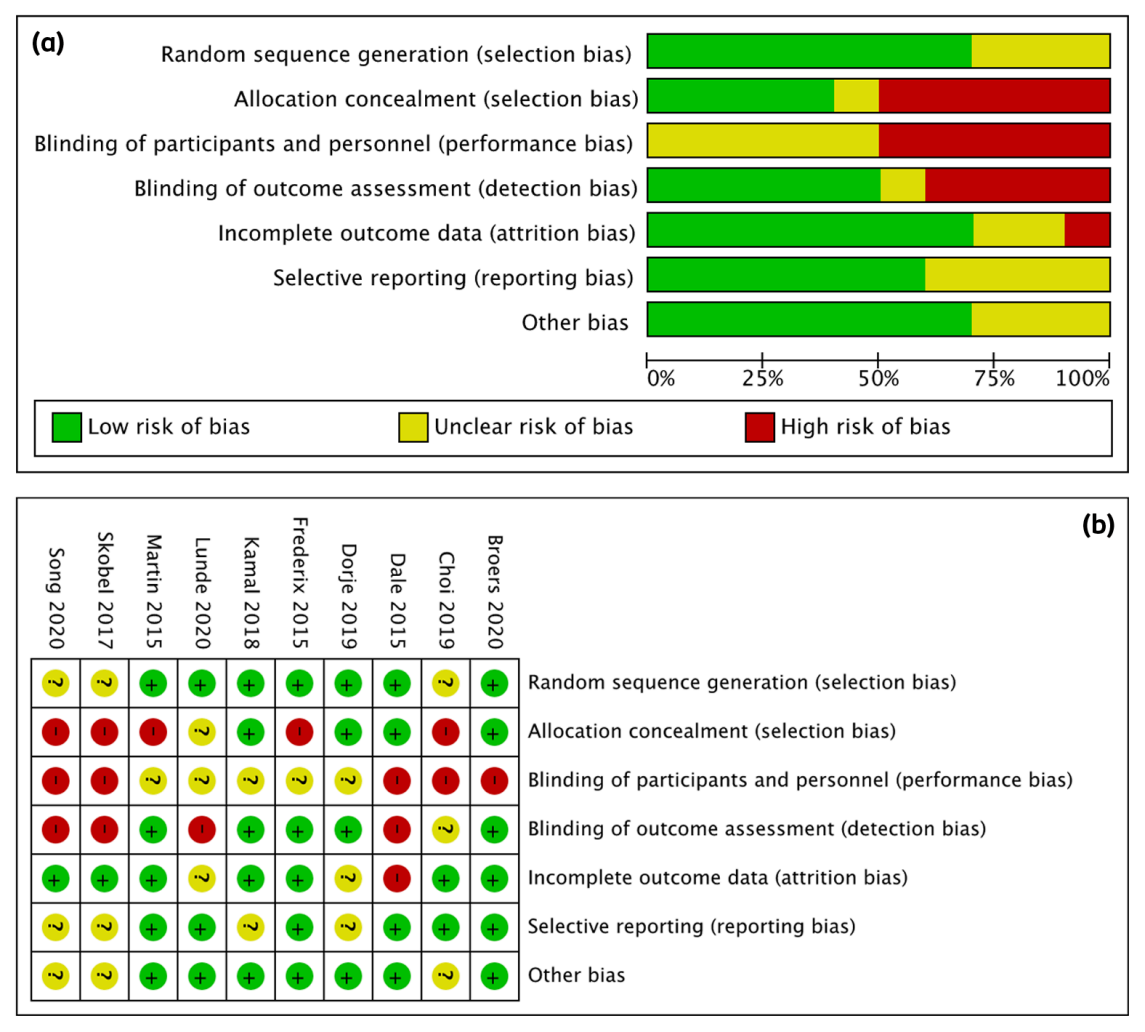
**(a)** The risk-of-bias graph: proportions of studies with low, high, and unclear risks of bias in each domain. **(b)** The risk-of-bias summary: the risk-of-bias judgement of each included study in each domain.

Derived from the 10 studies included in this review, Figure 2a depicts the proportion of studies with low, high, and unclear RoB in each domain. In contrast, Figure 2b illustrates the RoB judgement for each included study in each domain. Overall, the studies’ RoB varied significantly across domains, with the majority assessed to be low risks in random sequence generation, incomplete outcome data, selective reporting, and a minority were evaluated to be low risks in allocation concealment. A considerable proportion of studies were discovered to have a high RoB in allocation concealment, blinding of participants, personnel and outcome assessment, as well as, incomplete outcome data. Meanwhile, a sizeable proportion of the studies included in this review lacked sufficient information to permit a meaningful RoB assessment.

### 3.2 Physical health outcomes

The effect of eHealth interventions on physical health outcomes assessed were BMI (body mass index), systolic and diastolic resting blood pressure (BP), and lipid profile (low density lipoprotein (LDL), high density lipoprotein (HDL) and total cholesterol). In total, six studies [19,20,22,24,25,27] with 895 participants (441 patients who received eHealth interventions and 160 controls) contributed data, while the outcome data of four studies [21,23,26,28] were not reported sufficiently for meta-analysis.

Supplementary Document 6 contains a complete list of the physical health outcomes estimates for each comparison. For certainty-of-evidence ratings of the physical health outcomes, and reasons for downgrading, see the Summary of findings (Supplementary Document 4).

#### 1. Body Mass Index

**Figure 3:**
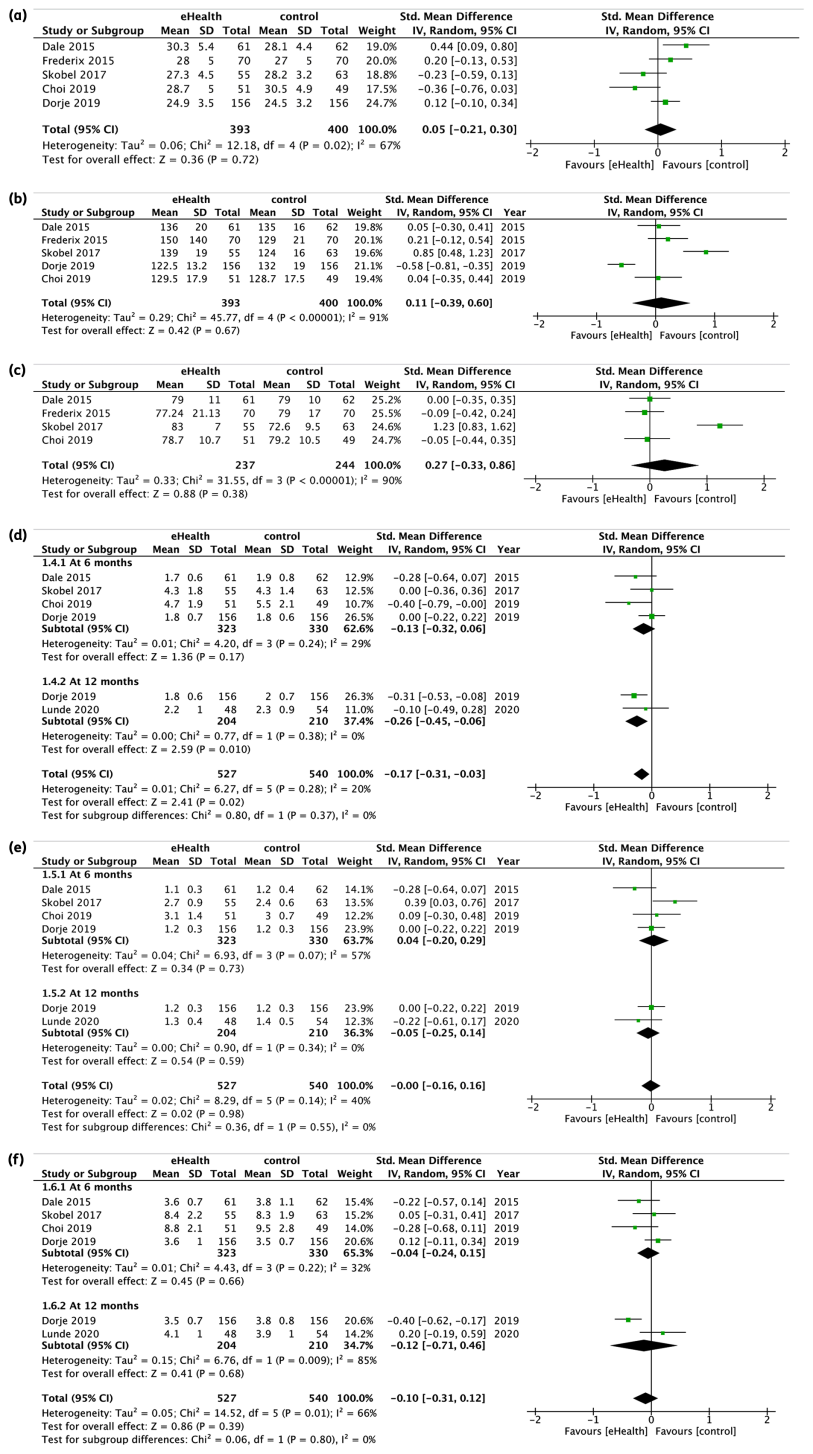
The effect of eHealth intervention on physical health outcomes. **(a)** The effect of eHealth intervention on BMI at 6 months (n = 5). **(b)** The effect of eHealth intervention on Systolic BP at 6 months (n = 5). **(c)** The effect of eHealth intervention on Diastolic BP at 6 months (n = 4). **(d)** The effect of eHealth intervention on LDL at 6 and 12 months (n = 6). **(e)** The effect of eHealth intervention on HDL at 6 and 12 months (n = 6). **(f)** The effect of eHealth intervention on Total Cholesterol at 6 and 12 months (n = 6).

BMI refers to the change of participants’ body mass index at six months in kilogram per metre squared (kg/m^2^). Data from five studies were pooled and indicated that eHealth interventions did not improve BMI, however it was not significant (SMD 0.05, 95% CI [–0.21, 0.30], I^2^ = 67%, p = 0.72; Figure 3a).

#### 2. Blood Pressure

BP refers to the change of resting BP in terms of systolic and diastolic at six months in millimetres of mercury (mmHg). Data from five studies for systolic and four studies for diastolic were pooled and indicated that eHealth interventions did not significantly improve systolic (SMD 0.11, 95% CI [–0.39, 0.60], I^2^ = 91%, p = 0.67; Figure 3b) and diastolic (SMD 0.27, 95% CI [–0.33, 0.86], I^2^ = 90%, p = 0.38; Figure 3c).

#### 3. Lipid profile

The effect of eHealth interventions on LDL was evaluated at 6 and 12 months in terms of milimoles per litre (mmol/L). Due to the substantial degree of heterogeneity in the pooled results of the four included studies, they were divided into subgroups according to the timing of outcome measurement (at 6 and 12 months). A difference in LDL levels favouring eHealth interventions was found. eHealth interventions have significantly improved LDL at 12 months (SMD -0.26, 95% CI [–0.45, -0.06], I^2^ = 0%, p = 0.01), but not at 6 months (SMD -0.13, 95% CI [–0.32, 0.06], I^2^ = 29%, p = 0.17; Figure 3d).

Meanwhile, the effect of eHealth interventions on HDL was evaluated via subgroup analysis at 6 and 12 months (mmol/L), but did not show significant differences (SMD 0.04, 95% CI [–0.20, 0.29], I^2^ = 57%, p = 0.73) at 6 months and at 12 months (SMD -0.05, 95% CI [–0.25, 0.14], I^2^ = 0%, p = 0.34; Figure 3e).

Lastly, the effect of eHealth interventions on total cholesterol was evaluated via subgroup analysis at 6 and 12 (mmol/L), but did not show significant differences (SMD -0.04, 95% CI [–0.24, 0.15], I^2^ = 32%, p = 0.66) at 6 months and (SMD -0.12, 95% CI [–0.71, 0.46], I^2^ = 85%, p = 0.68) at 12 months (Figure 3f).

**Figure 4:**
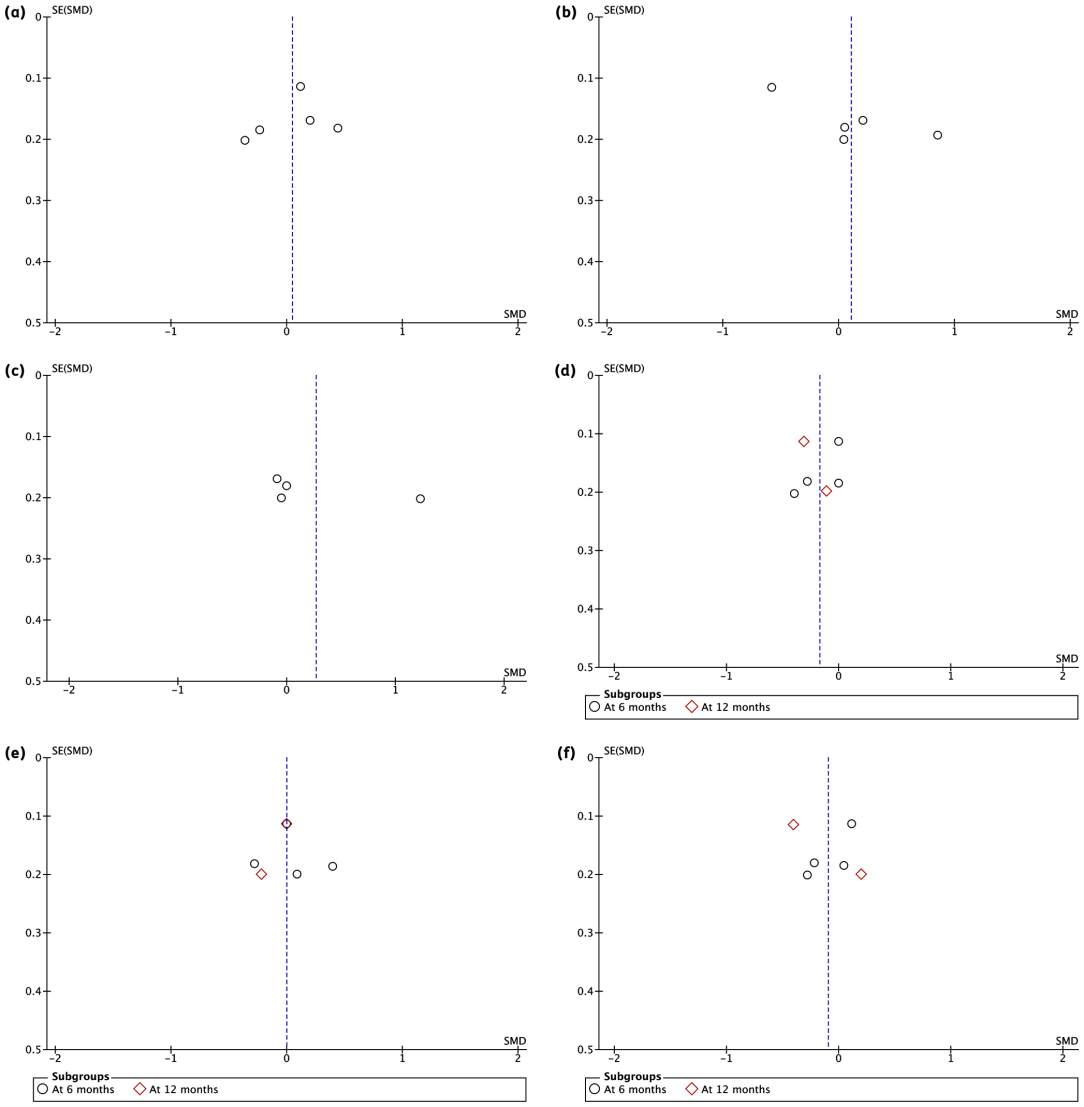
Funnel plot comparison of eHealth intervention for physical health outcomes. **(a)** Funnel plot comparison of eHealth intervention for BMI (n = 5). **(b)** Funnel plot comparison of eHealth intervention for resting systolic BP at 6 months (n = 5). **(c)** Funnel plot comparison of eHealth intervention for resting diastolic BP at 6 months (n = 4). **(d)** Funnel plot comparison of eHealth intervention for LDL at 6 and 12 months (n = 6). **(e)** Funnel plot comparison of eHealth intervention for HDL at 6 and 12 months (n = 6). **(f)** Funnel plot comparison of eHealth intervention for Total Cholesterol at 6 and 12 months (n = 6).

Overall, the funnel plots generated were asymmetrical due to differences among the studies (Figure 4).

#### 3.2.1 Sensitivity Analysis

**Figure 5:**
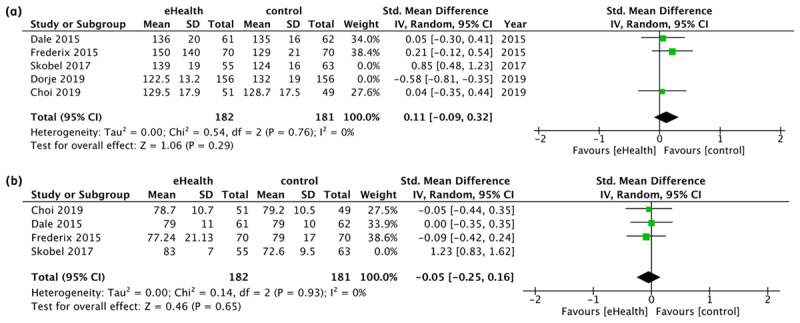
Sensitivity analysis on the effect of eHealth intervention on BP at 6 months. **(a)** Sensitivity analysis on the effect of eHealth intervention on Systolic BP at 6 months (n = 3). **(b)** Sensitivity analysis on the effect of eHealth intervention on Diastolic BP at 6 months (n = 3).

From the analysis of physical health outcomes, based on the sensitivity analysis of those with significant heterogeneity, the effectiveness of eHealth interventions might be related to the differences in target population, race or ethnicity, as Skobel 2017 [22] is a multi-centre study and Dorje 2019 [25] is a study conducted among the Chinese. By excluding these studies from the meta-analysis, the heterogeneity improved for BP effect estimates for systolic (SMD 0.11, 95% CI [–0.09, 0.32], I^2^ = 0%, p = 0.29; Figure 5a) and diastolic (SMD –0.05, 95% CI [–0.25, 0.16], I^2^ = 0%, p = 0.65; Figure 5b). Nevertheless, the results were still not significant.

### 3.3 Non-physical health outcomes

The utilisation of eHealth interventions can be seen from the domains derived for non-physical health outcomes, which are: (1) Behavioural, (medication adherence, physical activity, dietary behaviour and others); (2) Psychological, (anxiety and depression); and (3) other health outcomes (QoL). For non-physical health outcomes, instead of meta-analysis, synthesis without meta-analysis (SWiM) was performed in view of data variability with various scale measurements utilised and different duration outcomes. The final outcome summary deduced from all 10 studies was categorised accordingly (Table 3). Significance appraisal between comparable papers in each domain found half of the significant findings were medication adherence, physical activity and dietary behaviour, and half of the non-significant findings were other behavioural outcomes, psychological and QoL.

**Table 3:** Outcome summary for non-physical health outcomes utilising eHealth interventions (n = 10).


AUTHOR, YEAR, STUDY DURATION OUTCOME	NON-PHYSICAL HEALTH OUTCOME (EHEALTH INTERVENTION VERSUS CONTROL)					

	BEHAVIOURAL				PSYCHOLOGICAL	QUALITY OF LIFE

	MEDICATION ADHERENCE	PHYSICAL ACTIVITY	DIETARY BEHAVIOUR	OTHERS		

Dale 2015 [19], at 6 months	MMAS-8 Mean difference: 0.58, 95% CI 0.19–0.97; ** p = 0.004	Godin Leisure Time Physical Activity Questionnaire IG: 28% to 31% CG: 11% to 24%	–	Self-reported composite health behaviour AOR: 1.93, 95% CI 0.83–4.53; p = 0.13 IG: 33% to 53%CG: 27% to 39%	HADS (anxiety) Mean difference: 1.18, 95% CI 0.28, 2.08; * p = 0.01	–

Frederix 2015 [20], at 6 months	–	IPAQ in MET-min/week of VMW IG: χ2 (2): 13.7; * p = 0.01CG: χ2 (2): 0.6; p = 0.72	–	–	–	HRQL IG: χ2 (2): 14.0; ** p < 0.001 CG: χ2 (2): 3.1; p = 0.21

Martin 2015 [21]	–	Daily steps Mean difference: 3376, 95% CI 1951–4801; ** p < 0.001	–	–	–	–

Skobel 2017 [22], at 6 months	–	–	–	–	HADS (Anxiety) Mean (SD); p = 0.1 IG: 1.6 (3.1) CG: –0.63 (3.8) HADS (Depression) Mean (SD) p = 0.27 IG: 1.36 (3.7) CG: 0 (1.6)	EQ–5D Mean (SD); p = 0.98 IG: 0.64 (13.9) CG: 0.54 (10.7)

Kamal 2018 [23], at 3 months	MMAS–8 Mean difference (SD): –0.06 (0.13), 95% CI –0.39–0.19; p = 0.69	–	–	–	–	–

Choi 2019 [24], at 6 months	–	–	MDS compliance (** p < 0.001) IG: 27.5% to 64.7% CG: 18.4% to 57.1%	–	–	–

Dorje 2019 [25], at 6 months	Adherence to cardioprotective medications Mean difference: 1.79, 95% CI 1.76–1.87; * p = 0.019	SF-12 Physical Health score Mean difference: 1.26, 95% CI –0.74 – 3.26; p = 0.22	–	Smoking status Mean difference: 2.42, 95% CI 0.42–14.00; p = 0.32	GAD–7 Mean difference: 0.60, 95% CI –0.25 – 1.46; p = 0.17	–

Broers 2020 [26], at 6 months	–	–	–	Health Promotion Lifestyle Profile F _unadjusted_ (2,271.90) = 8.28; ** p < 0.001	–	WHOQOL-BREF F(2,135.42) = 9.63; ** p < 0.001

Lunde 2020 [27], at 1 year	–	Exercise habits Mean change (SD) IG: 1.4 (1.5); (** p < 0.001) CG: 0.6 (1.1); (** p < 0.001)	–	–	–	Heart QoL Mean change (SD) IG: 0.21 (0.47); (* p < 0.05) CG: 0.09 (0.45)

Song 2020 [28], at 6 months	–	Exercise habits based on ACSM χ2 9.826; * p = 0.02	–	–	-	–

**Outcome summary**	**Significant**	**Significant**	**Significant**	**Not significant**	**Not significant**	**Not significant**


IG: Intervention group, CG: Control group, MMAS-8: Morisky 8-item Medication Adherence Questionnaire, HADS: Hospital Anxiety and Depression Scale, VMW: vigorous and/or moderate and/or walking, IPAQ: International Physical Activity Questionnaire, HRQL: Health-Related Quality of Life, χ2 (2): Friedman’s test, EQ-5D: European Quality of Life Five Dimension, MDS: Mediterranean diet scores, SF-12: 12-Item Short Form Survey, GAD-7: GAD-7: Generalised Anxiety Disorder 7-item scale, WHOQOL-BREF: World Health Organization Quality of Life–BREF, F: Analysis of Variance (ANOVA), HeartQol: Heart Quality of Life, ACSM: 10^th^ Edition American College of Sports Medicine Guidelines for Exercise Testing and Prescription. * Statistical significance: p < 0.05, ** p < 0.005.

## 4.0 Discussion

The review determined that eHealth interventions have significant effects on LDL in patients with IHD but had non-significant effects on other health outcomes. The eHealth interventions were used in secondary prevention to monitor patients’ vital signs and lifestyle modification adherence, and hence, improved their overall QoL.

Here, the main finding demonstrated that 12-month eHealth interventions improved LDL levels in IHD patients as compared to usual care. The meta-analysis demonstrated that a long-term and sustainable intervention is essential for reducing LDL. Supported by current IHD treatment guidelines, LDL remains one of the primary treatment target for reducing ischaemic events and in secondary prevention follow-up visits, where lower levels are better [34,35]. Notably, LDL particles act as a major cholesterol transporter and are the key contributor to the increased risk of atherosclerotic lesion formation [36]. Similarly, elevated HDL is correlated with decreased risk in atherosclerosis from epidemiological studies but yielded null results in therapeutic clinical trials [37]. This justification was reflected in the non-significant findings for HDL and total cholesterol, as LDL largely replaced total cholesterol as a risk marker and primary treatment target [38]. Long-term interventions require adherence to both pharmacotherapy and non-pharmacotherapy methods by applying modalities such as mobile smartphone apps and SMS [25,27]. The results also suggested that eHealth interventions implemented heterogeneously across countries and populations were successful, which was supported by moderate to high GRADE effect estimates (Supplementary Document 4).

Other physical health outcomes (BMI, BP, HDL, total cholesterol) exerted no significant effects with eHealth interventions, even with sensitivity analysis of BP findings to improve heterogeneity due to population differences. This is possibly reflected by the small sample size and high RoB (Figure 2a, 2b) from allocation concealment and participant and personnel blinding. In some studies, the differences in the technologies used in eHealth interventions and the intervention duration, e.g. 6 months or 12 months, might be insufficient to improve certain cardio-metabolic parameters. Furthermore, a limited number of studies were able to explain the non-significant changes. Here, we included a total of six RCTs; therefore, additional research is required to confirm the results. Moreover, the asymmetrical shapes of the overall funnel plots suggested the existence of publication bias, which is induced because statistically significant results are more likely to be published than null or non-significant results. Hence, publication bias may threaten the validity of such analyses, leading to incorrect, typically over-optimistic conclusions [39].

The findings for the non-physical health outcome domains measured in this study revealed that subjective measurements are ambiguous and how patients benefit from eHealth interventions can be interpretd differently. The beneficial effects of eHealth interventions can sustain lifestyle modification (medication adherence, diet, and exercise) via constant professional support and individualised lifestyle behavioural changes [11]. However, high-quality evidence is lacking and most evidence was from high-income countries [40], where most studies [19,20,21,22,24,26,27,40] in the present review were conducted in Europe or North America.

The non-significant finding of psychological health outcomes and QoL revealed that the ambiguous effects of eHealth interventions identified in this review might be related and linked to the underpinning health behaviour theory: the Transactional Model of eHealth Literacy (TMeHL). Transactional Model of eHealth Literacy is a continuous communication transaction process that is constantly modified according to eHealth contextual factors and prior eHealth experiences. Based on the seminal component of eHealth literacy levels, the interplay between task-oriented factors (usability of eHealth interventions modality) and user-oriented factors (age, race) would result in health outcomes by empowering patients with eHealth skills [41]. The present review demonstrated the user-oriented factor in certain studies with a mean participant age of >60 years [20,22,25,26]. Older people, who typically have deteriorating cognitive ability and memory, lack effective learning methods for advancement via mobile devices and limited Internet applications and exposure [42]. Coupled with the task-oriented factors of various modalities used in the present study, the included studies captured from 2015 to 2020 revealed the evolution of various eHealth interventions used to influence the non-significance of the non-physical health outcome results. Older adults may be less familiar with novel eHealth technologies and thus struggle to accept and adapt to modern technological devices; accordingly, there should be special consideration of the receptivity, memory, and auditory abilities of the elderly [43]. Nonetheless, none of the included studies mentioned the fundamental eHealth literacy level or other literacy levels of its kind. These literacy levels are essential as health experts have concluded that patients’ knowledge or literacy levels for compatible use of eHealth devices with their users should be assessed to produce excellent health outcomes [44].

Despite physical health outcomes being measured in a standardised and universally accepted method, cautious interpretation is vital. Therefore, we conclude that eHealth interventions can improve the long-term LDL for IHD patients. Further randomised trials with adequate blinding and longer-term follow-up may demonstrate that eHealth interventions have better and significant health outcomes. Hence, eHealth interventions are useful for enhancing specific biomarker results. Researchers or physicians should determine whether certain eHealth interventions are appropriate based on their study objective and their patients’ needs. The findings also suggested that future theory-guided trial interventions are needed as is the need to consider other health behaviour moderators and mediators. Issues aligned with the TMeHL theory might explain the non-significant findings that may be incorporated in providing IHD patients with corresponding eHealth modalities that match their eHealth literacy levels and task- and user-oriented factors. This finding is in accordance with the simple mHealth group intervention strategy that renders health information delivered via SMS, WeChat, and email more acceptable to older adults [43]. Empirical studies supported using any kind of eHealth interventions to improve patients’ health outcomes for various non-communicable diseases, for example, cancer [45,46,47], diabetes [48,49,50] and predominantly IHD [51,52,53,54].

IHD treatment also includes various target components and combinations. However, there are no specific interventions for patients with various cardio-metabolic components. Therefore, it is necessary to optimise the rapidly developing eHealth interventions to provide precise care to patients with IHD. Following the pathways in developed and developing nations, the implementation could bes advantageous for healthcare providers providing recommendations for patients with distinct characteristics. In addition to governance and regulatory challenges, the remaining hurdles are information management, interoperability, and integration. These hurdles include the capacity to enable communication technologies and the availability of online information for doctors and patients with IHD that can help manage co-morbidities [55]. Moreover, the findings were generated from a small number of RCTs and we believe that studies involving more regions and larger samples are required before eHealth interventions may be suggested in future guidelines [56].

### 4.1 Strengths and Limitations

To our knowledge, this is the first meta-analysis that focused on the health outcomes of IHD patients by utilising eHealth interventions. The findings are relevant regarding IHD outcomes achieved through the suitability of eHealth interventions that address the unique healthcare needs of IHD patients considering age, literacy level, and population.

Only three studies were conducted in Asia, while the remaining studies were conducted in Europe or North America, which limited the generalisability of the results. Further eHealth interventions with rigorous study designs and more diverse populations from different cultural contexts are required to produce credible evidence. Most of the included studies were pilot and feasibility studies with different durations and used a broad range of eHealth interventions. However, patients may be sceptical of new technology for various reasons, including their preconceptions or unfamiliarity with the use of that technology, which can affect user satisfaction and long-term intervention adherence. Thus, the future challenges for researchers and clinicians are to design studies that incorporate patient preferences and to significantly improve intervention study reporting. These aspects are critical so that clinicians and researchers can assess the feasibility of implementing eHealth interventions for patient education and secondary prevention not only in cardiac care but also in other patient groups. Additionally, the participants’ eHealth literacy levels and computer literacy skills are unknown, which may have influenced the study outcomes. As the reviewed RCTs typically featured inadequate concealment and blinding, evaluating their methodological rigour was challenging, which resulted in a high risk of selection and performance bias. Additional studies should be conducted to strengthen concealment and outcome reporting methods to improve the quality of evidence.

## 5.0 Conclusion

Based on moderate to high effect estimates, eHealth interventions in long-term could effectively lower LDL cholesterol. However, given the study limitations, the effects of eHealth interventions on other physical and non-physical health outcomes remain inconclusive. It is recommended that sustainable patient empowerment strategies be integrated with the advancement of eHealth interventions for future research by utilising appropriate frameworks considering other potential health behaviour moderators and mediators.

## Data Accessibility Statements

The dataset used and analysed during this review is available from the corresponding author on reasonable request.

## Additional File

The additional file for this article can be found as follows:

10.5334/gh.1173.s1 Supplementary Documents. Supplementary Documents 1 to 6.
