# Supplementary material for: A Meta-Analysis of eHealth Interventions on Ischaemic Heart Disease Health Outcomes
Source: Glob Heart. 2023 Mar 16;18(1):12. doi: 10.5334/gh.1173 (PMC10022534; doi:10.5334/gh.1173)
Supplement: Supplementary Documents. — Supplementary Documents 1 to 6. [file gh-18-1-1173-s1.pdf]

SUPPLEMENTARY DOCUMENT 1

PRISMA 2020 Checklist

Section and Topic	Item #	Checklist item	Location where item is reported (Page)
**TITLE**			
Title	1	Identify the report as a systematic review.	1
**ABSTRACT**			
Abstract	2	See the PRISMA 2020 for Abstracts checklist.	2
**INTRODUCTION**			
Rationale	3	Describe the rationale for the review in the context of existing knowledge.	4
Objectives	4	Provide an explicit statement of the objective(s) or question(s) the review addresses.	6
**METHODS**			
Eligibility criteria	5	Specify the inclusion and exclusion criteria for the review and how studies were grouped for the syntheses.	8
Information sources	6	Specify all databases, registers, websites, organisations, reference lists, and other sources searched or consulted to identify studies. Specify the date when each source was last searched or consulted.	7
Search strategy	7	Present the entire search strategies for all databases, registers and websites, including any filters and limits used.	Supplementary Document 3
Selection process	8	Specify the methods used to decide whether a study met the inclusion criteria of the review, including how many reviewers screened each record and each report retrieved, whether they worked independently, and if applicable, details of automation tools used in the process.	8
Data collection process	9	Specify the methods used to collect data from reports, including how many reviewers collected data from each report, whether they worked independently, any processes for obtaining or confirming data from study investigators, and if applicable, details of automation tools used in the process.	8
Data items	10a	List and define all outcomes for which data were sought. Specify whether all results that were compatible with each outcome domain in each study were sought (e.g. for all measures, time points, analyses), and if not, the methods used to decide which results to collect.	8
	10b	List and define all other variables for which data were sought (e.g. participant and intervention characteristics, funding sources). Describe any assumptions made about any missing or unclear information.	8
Study risk of bias assessment	11	Specify the methods used to assess the risk of bias in the included studies, including details of the tool(s) used, how many reviewers assessed each study and whether they worked independently, and if applicable, details of automation tools used in the process.	9
Effect measures	12	Specify for each outcome the effect measure(s) (e.g. risk ratio, mean difference) used in the synthesis or presentation of results.	10
Synthesis methods	13a	Describe the processes used to decide which studies were eligible for each synthesis (e.g. tabulating the study intervention characteristics and comparing	11

Section and Topic	Item #	Checklist item	Location where item is reported (Page)
		against the planned groups for each synthesis (item #5)).	
	13b	Describe any methods required to prepare the data for presentation or synthesis, such as handling of missing summary statistics or data conversions.	10
	13c	Describe any methods used to tabulate or visually display the results of individual studies and syntheses.	10
	13d	Describe any methods used to synthesise results and provide a rationale for the choice(s). If meta-analysis was performed, describe the model(s), method(s) to identify the presence and extent of statistical heterogeneity, and software package(s) used.	10
	13e	Describe any methods used to explore possible causes of heterogeneity among study results (e.g. subgroup analysis, meta-regression).	10
	13f	Describe any sensitivity analyses conducted to assess the robustness of the synthesised results.	10
Reporting bias assessment	14	Describe any methods used to assess the risk of bias due to missing results in a synthesis (arising from reporting biases).	9
Certainty assessment	15	Describe any methods used to assess certainty (or confidence) in the body of evidence for an outcome.	10
**RESULTS**			
Study selection	16a	Describe the results of the search and selection process, from the number of records identified in the search to the number of studies included in the review, ideally using a flow diagram.	8
	16b	Cite studies that might appear to meet the inclusion criteria but which were excluded, and explain why they were excluded.	8-9
Study characteristics	17	Cite each included study and present its characteristics.	11
Risk of bias in studies	18	Present assessments of risk of bias for each included study.	11
Results of individual studies	19	For all outcomes, present, for each study: (a) summary statistics for each group (where appropriate) and (b) an effect estimate and it's precision (e.g. confidence/credible interval), ideally using structured tables or plots.	12-14
Results of syntheses	20a	For each synthesis, briefly summarise the characteristics and risk of bias among contributing studies.	11-13
	20b	Present results of all statistical syntheses conducted. If meta-analysis was done, present for each the summary estimate and its precision (e.g. confidence/credible interval) and measures of statistical heterogeneity. If comparing groups, describe the direction of the effect.	16
	20c	Present results of all investigations of possible causes of heterogeneity among study results.	16
	20d	Present results of all sensitivity analyses conducted to assess the robustness of the synthesised results.	16
Reporting biases	21	Present assessments of risk of bias due to missing results (arising from reporting biases) for each synthesis assessed.	-
Certainty of	22	Present assessments of certainty (or confidence) in the body of evidence for each outcome assessed.	Supplementary

Section and Topic	Item #	Checklist item	Location where item is reported (Page)
evidence			Document 4
**DISCUSSION**			
Discussion	23a	Provide a general interpretation of the results in the context of other evidence.	17, 21
	23b	Discuss any limitations of the evidence included in the review.	22
	23c	Discuss any limitations of the review processes used.	22
	23d	Discuss implications of the results for practice, policy, and future research.	21-23
**OTHER INFORMATION**			
Registration and protocol	24a	Provide registration information for the review, including register name and registration number, or state that the review was not registered.	7
	24b	Indicate where the review protocol can be accessed or state that a protocol was not prepared.	7
	24c	Describe and explain any amendments to the information provided at registration or in the protocol.	-
Support	25	Describe sources of financial or non-financial support for the review and the role of the funders or sponsors in the review.	24
Competing interests	26	Declare any competing interests of review authors.	24
Availability of data, code and other materials	27	Report which of the following are publicly available and where they can be found: template data collection forms; data extracted from included studies; data used for all analyses; analytic code; any other materials used in the review.	24

SUPPLEMENTARY DOCUMENT 2

PRISMA 2020 for Abstracts Checklist

Section and Topic	Item #	Checklist item	Reported (Yes/No)
**TITLE**			
Title	1	Identify the report as a systematic review.	Yes
**BACKGROUND**			
Objectives	2	Provide an explicit statement of the main objective(s) or question(s) the review addresses.	Yes
**METHODS**			
Eligibility criteria	3	Specify the inclusion and exclusion criteria for the review.	Yes
Information sources	4	Specify the information sources (e.g. databases, registers) used to identify studies and the date when each was last searched.	Yes
Risk of bias	5	Specify the methods used to assess the risk of bias in the included studies.	Yes
Synthesis of results	6	Specify the methods used to present and synthesise results.	Yes
**RESULTS**			
Included studies	7	Give the total number of included studies and participants and summarise relevant characteristics of studies.	Yes
Synthesis of results	8	Present results for main outcomes, preferably indicating the number of included studies and participants for each. If meta-analysis was done, report the summary estimate and confidence/credible interval. If comparing groups, indicate the direction of the effect (i.e. which group is favoured).	Yes
**DISCUSSION**			
Limitations of evidence	9	Provide a brief summary of the limitations of the evidence included in the review (e.g. study risk of bias, inconsistency and imprecision).	Yes
Interpretation	10	Provide a general interpretation of the results and important implications.	Yes
**OTHER**			
Funding	11	Specify the primary source of funding for the review.	Yes

Section and Topic	Item #	Checklist item	Reported (Yes/No)
Registration	12	Provide the registered name and registration number.	Yes

SUPPLEMENTARY DOCUMENT 3

Search string used for all databases

Database	Search String
Web of Science	TS=("Adult" OR "Young Adult" OR "Middle Aged" OR "Man" OR "Woman" OR "Men" OR "Women" OR "Patient" OR "Person") AND TS=("Ischaemic Heart Disease" OR "Ischemic Heart Disease" OR "Coronary artery disease" OR "Coronary atherosclerosis" OR "Coronary arteriosclerosis" OR "Left main disease" OR "Left main coronary disease" OR "Left main coronary artery disease") AND TS=("eHealth Literacy" OR "patient education" OR "consumer ehealth information" OR "electronic education" OR "electronic information literacy" OR "patient medication knowledge" OR "information science" OR "communication" OR "ehealth cognition" OR "ehealth education" OR "ehealth enlightenment" OR "medical informatics" OR "eLearning" OR "electronic medical records" OR "telehealth" OR "telemedicine" OR "mHealth") AND TS=("level" OR "quantity" OR "amount" OR "extent" OR "measure" OR "degree" OR "magnitude" OR "proportion" OR "status" OR "rank" OR "grade") AND TS=("outcome" OR "result" OR "consequence" OR "conclusion" OR "effect" OR "sequel" OR "issue" OR "physical health outcome" OR "clinical parameters" OR "health status" OR "risk of other chronic diseases" OR "behavioural outcomes" OR "self management" OR "medication adherence" OR "advice adherence" OR "disease screening" OR "online health information seeking" OR "health-promoting behaviours" OR "psychological health outcomes" OR "depression" OR "anxiety" OR "health related quality of life" OR "health care utilisation" OR "health attitudes" OR "decision making" OR "physical state" OR "physical fitness" OR "lifestyle adherence" OR "mental health outcome" OR "health improvement")
Scopus	TITLE-ABS-KEY("Adult" OR "Young Adult" OR "Middle Aged" OR "Man" OR "Woman" OR "Men" OR "Women" OR "Patient" OR "Person") AND TITLE-ABS-KEY("Ischaemic Heart Disease" OR "Ischemic Heart Disease" OR "Coronary artery disease" OR "Coronary atherosclerosis" OR "Coronary arteriosclerosis" OR "Left main disease" OR "Left main coronary disease" OR "Left main coronary artery disease") AND TITLE-ABS-KEY("eHealth Literacy" OR "patient education" OR "consumer ehealth information" OR "electronic education" OR "electronic information literacy" OR "patient medication knowledge" OR "information science" OR "communication" OR "ehealth cognition" OR "ehealth education" OR "ehealth enlightenment" OR "medical informatics" OR "eLearning" OR "electronic medical records" OR "telehealth" OR "telemedicine" OR "mHealth") AND TITLE-ABS-KEY("level" OR "quantity" OR "amount" OR "extent" OR "measure" OR "degree" OR "magnitude" OR "proportion" OR "status" OR "rank" OR "grade") AND TITLE-ABS-KEY("outcome" OR "result" OR "consequence" OR "conclusion" OR "effect" OR "sequel" OR "issue" OR "physical health outcome" OR "clinical parameters" OR "health status" OR "risk of other chronic diseases" OR "behavioural outcomes" OR "self management" OR "medication adherence" OR "advice adherence" OR "disease screening" OR "online health information seeking" OR "health-promoting behaviours" OR "psychological health outcomes" OR "depression" OR "anxiety" OR

	"health related quality of life" OR "health care utilisation" OR "health attitudes" OR "decision making" OR "physical state" OR "physical fitness" OR "lifestyle adherence" OR "mental health outcome" OR "health improvement")
PubMed	(((("Adult"[Title/Abstract] OR "Young Adult"[Title/Abstract] OR "Middle Aged"[Title/Abstract] OR "Man"[Title/Abstract] OR "Woman"[Title/Abstract] OR "Men"[Title/Abstract] OR "Women"[Title/Abstract] OR "Patient"[Title/Abstract] OR "Person"[Title/Abstract]) AND ("Adult"[Title/Abstract] OR "Young Adult"[Title/Abstract] OR "Middle Aged"[Title/Abstract] OR "Man"[Title/Abstract] OR "Woman"[Title/Abstract] OR "Men"[Title/Abstract] OR "Women"[Title/Abstract] OR "Patient"[Title/Abstract] OR "Person"[Title/Abstract])) AND ("eHealth Literacy"[Title/Abstract] OR "patient education"[Title/Abstract] OR "consumer ehealth information"[Title/Abstract] OR "electronic education"[Title/Abstract] OR "electronic information literacy"[Title/Abstract] OR "patient medication knowledge"[Title/Abstract] OR "information science"[Title/Abstract] OR "communication"[Title/Abstract] OR "ehealth cognition"[Title/Abstract] OR "ehealth education"[Title/Abstract] OR "ehealth enlightenment"[Title/Abstract] OR "medical informatics"[Title/Abstract] OR "eLearning"[Title/Abstract] OR "electronic medical records"[Title/Abstract] OR "telehealth"[Title/Abstract] OR "telemedicine"[Title/Abstract] OR "mHealth"[Title/Abstract])) AND ("level"[Title/Abstract] OR "quantity"[Title/Abstract] OR "amount"[Title/Abstract] OR "extent"[Title/Abstract] OR "measure"[Title/Abstract] OR "degree"[Title/Abstract] OR "magnitude"[Title/Abstract] OR "proportion"[Title/Abstract] OR "status"[Title/Abstract] OR "rank"[Title/Abstract] OR "grade"[Title/Abstract])) AND ("outcome"[Title/Abstract] OR "result"[Title/Abstract] OR "consequence"[Title/Abstract] OR "conclusion"[Title/Abstract] OR "effect"[Title/Abstract] OR "sequel"[Title/Abstract] OR "issue"[Title/Abstract] OR "physical health outcome"[Title/Abstract] OR "clinical parameters"[Title/Abstract] OR "health status"[Title/Abstract] OR "risk of other chronic diseases"[Title/Abstract] OR "behavioural outcomes"[Title/Abstract] OR "self management"[Title/Abstract] OR "medication adherence"[Title/Abstract] OR "advice adherence"[Title/Abstract] OR "disease screening"[Title/Abstract] OR "online health information seeking"[Title/Abstract] OR "health-promoting behaviours"[Title/Abstract] OR "psychological health outcomes"[Title/Abstract] OR "depression"[Title/Abstract] OR "anxiety"[Title/Abstract] OR "health related quality of life"[Title/Abstract] OR "health care utilisation"[Title/Abstract] OR "health attitudes"[Title/Abstract] OR "decision making"[Title/Abstract] OR "physical state"[Title/Abstract] OR "physical fitness"[Title/Abstract] OR "lifestyle adherence"[Title/Abstract] OR "mental health outcome"[Title/Abstract] OR "health improvement"[Title/Abstract])
EBSCOHost	SU ("Adult" OR "Young Adult" OR "Middle Aged" OR "Man" OR "Woman" OR "Men" OR "Women" OR "Patient" OR "Person") AND SU ("Ischaemic Heart Disease" OR "Ischemic Heart Disease" OR "Coronary artery disease" OR "Coronary atherosclerosis" OR "Coronary arteriosclerosis" OR "Left main disease" OR "Left main coronary disease" OR "Left main coronary artery disease") AND SU ("eHealth Literacy" OR "patient education" OR "consumer ehealth information" OR "electronic education" OR "electronic information literacy" OR "patient medication knowledge" OR "information science" OR "communication" OR "ehealth cognition"

	OR "ehealth education" OR "ehealth enlightenment" OR "medical informatics" OR "eLearning" OR "electronic medical records" OR "telehealth" OR "telemedicine" OR "mHealth") AND SU ("level" OR "quantity" OR "amount" OR "extent" OR "measure" OR "degree" OR "magnitude" OR "proportion" OR "status" OR "rank" OR "grade") AND SU ("outcome" OR "result" OR "consequence" OR "conclusion" OR "effect" OR "sequel" OR "issue" OR "physical health outcome" OR "clinical parameters" OR "health status" OR "risk of other chronic diseases" OR "behavioural outcomes" OR "self management" OR "medication adherence" OR "advice adherence" OR "disease screening" OR "online health information seeking" OR "health-promoting behaviours" OR "psychological health outcomes" OR "depression" OR "anxiety" OR "health related quality of life" OR "health care utilisation" OR "health attitudes" OR "decision making" OR "physical state" OR "physical fitness" OR "lifestyle adherence" OR "mental health outcome" OR "health improvement")
SAGE	[[Abstract "adult"] OR [Abstract "young adult"] OR [Abstract "middle aged"] OR [Abstract "man"] OR [Abstract "woman"] OR [Abstract "men"] OR [Abstract "women"] OR [Abstract "patient"] OR [Abstract "person"]] AND [[Abstract "ischaemic heart disease"] OR [Abstract "ischemic heart disease"] OR [Abstract "coronary artery disease"] OR [Abstract "coronary atherosclerosis"] OR [Abstract "coronary arteriosclerosis"] OR [Abstract "left main disease"] OR [Abstract "left main coronary disease"] OR [Abstract "left main coronary artery disease"]] AND [[Abstract "ehealth literacy"] OR [Abstract "patient education"] OR [Abstract "consumer ehealth information"] OR [Abstract "electronic education"] OR [Abstract "electronic information literacy"] OR [Abstract "patient medication knowledge"] OR [Abstract "information science"] OR [Abstract "communication"] OR [Abstract "ehealth cognition"] OR [Abstract "ehealth education"] OR [Abstract "ehealth enlightenment"] OR [Abstract "medical informatics"] OR [Abstract "elearning"] OR [Abstract "electronic medical records"] OR [Abstract "telehealth"] OR [Abstract "telemedicine"] OR [Abstract "mhealth"]] AND [[Abstract "level"] OR [Abstract "quantity"] OR [Abstract "amount"] OR [Abstract "extent"] OR [Abstract "measure"] OR [Abstract "degree"] OR [Abstract "magnitude"] OR [Abstract "proportion"] OR [Abstract "status"] OR [Abstract "rank"] OR [Abstract "grade"]] AND [[Abstract "outcome"] OR [Abstract "result"] OR [Abstract "consequence"] OR [Abstract "conclusion"] OR [Abstract "effect"] OR [Abstract "sequel"] OR [Abstract "issue"] OR [Abstract "physical health outcome"] OR [Abstract "clinical parameters"] OR [Abstract "health status"] OR [Abstract "risk of other chronic diseases"] OR [Abstract "behavioural outcomes"] OR [Abstract "self management"] OR [Abstract "medication adherence"] OR [Abstract "advice adherence"] OR [Abstract "disease screening"] OR [Abstract "online health information seeking"] OR [Abstract "health-promoting behaviours"] OR [Abstract "psychological health outcomes"] OR [Abstract "depression"] OR [Abstract "anxiety"] OR [Abstract "health related quality of life"] OR [Abstract "health care utilisation"] OR [Abstract "health attitudes"] OR [Abstract "decision making"] OR [Abstract "physical state"] OR [Abstract "physical fitness"] OR [Abstract "lifestyle adherence"] OR [Abstract "mental health outcome"] OR [Abstract "health improvement"]]

SUPPLEMENTARY DOCUMENT 4

Summary of findings tables with the certainty of evidence rating

Summary of findings:

eHealth Intervention compared to Control for ischaemic heart disease patients

Patient or population: ischaemic heart disease patients
Setting:
Intervention: eHealth Intervention
Comparison: Control

Outcomes	Anticipated absolute effects* (95% CI)		Relative effect (95% CI)	№ of participants (studies)	Certainty of the evidence (GRADE)	Comments
	**Risk with Control**	**Risk with eHealth Intervention**				
Body Mass Index (kg/m2) at 6 months	-	SMD **0.05 higher** (0.21 lower to 0.3 higher)	-	793 (5 RCTs)	⊕⊕⊕⊕ High[a,b]	
Resting Systolic BP at 6 months (mmHg)	-	SMD **0.11 higher** (0.39 lower to 0.6 higher)	-	793 (5 RCTs)	⊕⊕⊕◯ Moderate[a,c]	

Summary of findings:

eHealth Intervention compared to Control for ischaemic heart disease patients

Patient or population: ischaemic heart disease patients
Setting:
Intervention: eHealth Intervention
Comparison: Control

Outcomes	Anticipated absolute effects* (95% CI)		Relative effect (95% CI)	№ of participants (studies)	Certainty of the evidence (GRADE)	Comments
	Risk with Control	Risk with eHealth Intervention				
Resting Diastolic BP at 6 months	-	SMD **0.27 higher** (0.33 lower to 0.86 higher)	-	481 (4 RCTs)	⊕⊕⊕◯ Moderate[a,c]	
LDL (mmol/L)	-	SMD **0.17 lower** (0.31 lower to 0.03 lower)	-	1067 (5 RCTs)	⊕⊕⊕⊕ High[d]	
LDL (mmol/L) - At 6 months	-	SMD **0.13 lower** (0.32 lower to 0.06 higher)	-	653 (4 RCTs)	⊕⊕⊕⊕ High[d]	

Summary of findings:

eHealth Intervention compared to Control for ischaemic heart disease patients

Patient or population: ischaemic heart disease patients
Setting:
Intervention: eHealth Intervention
Comparison: Control

Outcomes	Anticipated absolute effects* (95% CI)		Relative effect (95% CI)	№ of participants (studies)	Certainty of the evidence (GRADE)	Comments
	**Risk with Control**	**Risk with eHealth Intervention**				
LDL (mmol/L) - At 12 months	-	SMD **0.26 lower** (0.45 lower to 0.06 lower)	-	414 (2 RCTs)	⊕⊕⊕⊕ High	
HDL (mmol/L)	-	SMD **0** (0.16 lower to 0.16 higher)	-	1067 (5 RCTs)	⊕⊕⊕⊕ High[d]	
HDL (mmol/L) - At 6 months	-	SMD **0.04 higher** (0.2 lower to 0.29 higher)	-	653 (4 RCTs)	⊕⊕⊕⊕ High[d]	

Summary of findings:

eHealth Intervention compared to Control for ischaemic heart disease patients

Patient or population: ischaemic heart disease patients
Setting:
Intervention: eHealth Intervention
Comparison: Control

Outcomes	**Anticipated absolute effects*** (95% CI)		Relative effect (95% CI)	№ of participants (studies)	Certainty of the evidence (GRADE)	Comments
	**Risk with Control**	**Risk with eHealth Intervention**				
HDL (mmol/L) - At 12 months	-	SMD **0.05 lower** (0.25 lower to 0.14 higher)	-	414 (2 RCTs)	⨁⨁⨁⨁ High	
Total Cholesterol (mmol/L)	-	SMD **0.1 lower** (0.31 lower to 0.12 higher)	-	1067 (5 RCTs)	⨁⨁⨁⨁ High[d]	
Total Cholesterol (mmol/L) - At 6 months	-	SMD **0.04 lower** (0.24 lower to 0.15 higher)	-	653 (4 RCTs)	⨁⨁⨁⨁ High[d]	

Summary of findings:

eHealth Intervention compared to Control for ischaemic heart disease patients

Patient or population: ischaemic heart disease patients
Setting:
Intervention: eHealth Intervention
Comparison: Control

Outcomes	**Anticipated absolute effects*** (95% CI)		Relative effect (95% CI)	№ of participants (studies)	Certainty of the evidence (GRADE)	Comments
	**Risk with Control**	**Risk with eHealth Intervention**				
Total Cholesterol (mmol/L) - At 12 months	-	SMD **0.12 lower** (0.71 lower to 0.46 higher)	-	414 (2 RCTs)	⨁⨁⨁⨁ High[b]	

***The risk in the intervention group** (and its 95% confidence interval) is based on the assumed risk in the comparison group and the **relative effect** of the intervention (and its 95% CI).

CI: confidence interval; **SMD:** standardised mean difference

Summary of findings:

eHealth Intervention compared to Control for ischaemic heart disease patients

Patient or population: ischaemic heart disease patients
Setting:
Intervention: eHealth Intervention
Comparison: Control

Outcomes	Anticipated absolute effects* (95% CI)		Relative effect (95% CI)	№ of participants (studies)	Certainty of the evidence (GRADE)	Comments
	**Risk with Control**	**Risk with eHealth Intervention**				

GRADE Working Group grades of evidence
High certainty: we are very confident that the true effect lies close to that of the estimate of the effect.
Moderate certainty: we are moderately confident in the effect estimate: the true effect is likely to be close to the estimate of the effect, but there is a possibility that it is substantially different.
Low certainty: our confidence in the effect estimate is limited: the true effect may be substantially different from the estimate of the effect.
Very low certainty: we have very little confidence in the effect estimate: the actual effect is likely to be substantially different from the estimate of effect.

Explanations

a. 3/5 studies had issues with allocation concealment and patient & personnel blinding
b. There was substantial heterogeneity, as indicated by a I^2 statistics
c. There was considerable heterogeneity. Sensitivity analysis revealed differences in population
d. 3/5 studies had issues with patient & personnel blinding and outcome blinding

SUPPLEMENTARY DOCUMENT 5

Synthesis Without Meta-analysis (SWiM) reporting items [29]

**SWiM is intended to complement and be used as an extension to PRISMA**			
**SWiM reporting item**	**Item description**	**Page in manuscript where item is reported**	**Other***
Methods			
**1** Grouping studies for synthesis	1a) Provide a description of, and rationale for, the groups used in the synthesis (e.g., groupings of populations, interventions, outcomes, study design)	10	
	1b) Detail and provide the rationale for any changes made subsequent to the protocol in the groups used in the synthesis	-	
**2** Describe the standardised metric and transformation methods used	Describe the standardised metric for each outcome. Explain why the metric(s) was chosen, and describe any methods used to transform the intervention effects, as reported in the study, to the standardised metric, citing any methodological guidance consulted	10	
**3** Describe the synthesis methods	Describe and justify the methods used to synthesise the effects for each outcome when it was not possible to undertake a meta-analysis of effect estimates	10	
**4** Criteria were used to prioritise results for summary and synthesis	Where applicable, provide the criteria used, with supporting justification, to select the particular studies, or a particular study, for the main synthesis or to conclude the synthesis (e.g., based on study design, risk of bias assessments, directness in relation to the review question)	10	

SWiM reporting item	Item description	Page in manuscript where item is reported	Other*
**5** Investigation of heterogeneity in reported effects	State the method(s) used to examine heterogeneity in reported effects when it was not possible to undertake a meta-analysis of effect estimates and its extensions to investigate heterogeneity	-	
**6** Certainty of evidence	Describe the methods used to assess the certainty of the synthesis findings	-	
**7** Data presentation methods	Describe the graphical and tabular methods used to present the effects (e.g., tables, forest plots, harvest plots).<br>Specify key study characteristics (e.g., study design, risk of bias) used to order the studies in the text and any tables or graphs, clearly referencing the studies included	17	
Results			
**8** Reporting results	For each comparison and outcome, provide a description of the synthesised findings and the certainty of the findings. Describe the result in language that is consistent with the question the synthesis addresses, and indicate which studies contribute to the synthesis	17, 37-39	
Discussion			
**9** Limitations of the synthesis	Report the limitations of the synthesis methods used and/or the groupings used in the synthesis and how these affect the conclusions that can be drawn in relation to the original review question	22	

PRISMA=Preferred Reporting Items for Systematic Reviews and Meta-Analyses.

SUPPLEMENTARY DOCUMENT 6

Summary of outcome estimates

eHealth intervention versus Control

Outcome or Subgroup	Studies	Participants	Statistical Method	Effect Estimate
1.1 Body Mass Index (kg/m2) at 6 months	5	793	Std. Mean Difference (IV, Random, 95% CI)	0.05 [−0.21, 0.30]
1.2 Resting Systolic BP at 6 months (mmHg)	5	793	Std. Mean Difference (IV, Random, 95% CI)	0.11 [−0.39, 0.60]
1.4 Resting Diastolic BP at 6 months	4	481	Std. Mean Difference (IV, Random, 95% CI)	0.27 [−0.33, 0.86]
1.5 LDL (mmol/L)	5	1067	Std. Mean Difference (IV, Random, 95% CI)	−0.17 [−0.31, −0.03]
1.5.1 At 6 months	4	653	Std. Mean Difference (IV, Random, 95% CI)	−0.13 [−0.32, 0.06]
1.5.2 At 12 months	2	414	Std. Mean Difference (IV, Random, 95% CI)	−0.26 [−0.45, −0.06]
1.6 HDL (mmol/L)	5	1067	Std. Mean Difference (IV, Random, 95% CI)	−0.00 [−0.16, 0.16]
1.6.1 At 6 months	4	653	Std. Mean Difference (IV, Random, 95% CI)	0.04 [−0.20, 0.29]
1.6.2 At 12 months	2	414	Std. Mean Difference (IV, Random, 95% CI)	−0.05 [−0.25, 0.14]
1.7 Total Cholesterol (mmol/L)	5	1067	Std. Mean Difference (IV, Random, 95% CI)	−0.10 [−0.31, 0.12]
1.7.1 At 6 months	4	653	Std. Mean Difference (IV, Random, 95% CI)	−0.04 [−0.24, 0.15]
1.7.2 At 12 months	2	414	Std. Mean Difference (IV, Random, 95% CI)	−0.12 [−0.71, 0.46]
